# A Non-Contact Method of Measuring Capillary Rise Based on the Hygroscopic Expansion of the Material

**DOI:** 10.3390/ma18153501

**Published:** 2025-07-25

**Authors:** Andrzej Kucharczyk, Kamil Pawlik, Mariusz Czabak

**Affiliations:** Faculty of Civil Engineering and Architecture, Opole University of Technology, Katowicka 48, 45-061 Opole, Poland; a.kucharczyk@po.edu.pl (A.K.); pawlik@po.edu.pl (K.P.)

**Keywords:** capillary water uptake, porous materials, moisture-induced expansion, non-contact measurement

## Abstract

This paper presents a novel, non-contact method for measuring capillary water uptake in porous materials based on the phenomenon of moisture-induced expansion. The proposed approach establishes a quantitative relationship between the amount of water absorbed by the material and the deformations measured on its surface. Digital Image Correlation (DIC) was used to track the displacements of reference points on gypsum specimens during capillary rise. The absorbed water mass was determined from the recorded displacements using a mechanical model that incorporates the moisture expansion coefficient. The method was validated by comparison with conventional continuous gravimetric measurements. The results demonstrate that the displacement-based approach accurately captures the capillary rise process, particularly in the initial phase, where the gravimetric method suffers from significant measurement errors due to surface tension effects. The proposed method eliminates these limitations, providing higher accuracy and temporal resolution. In addition, it enables the testing of larger samples and offers the potential for spatially resolved moisture analysis. The findings confirm that the method is suitable for studying moisture transport in porous materials and may serve as a valuable alternative to traditional gravimetric techniques.

## 1. Introduction

Capillary rise is an often-undesirable phenomenon in which water, as a result of contact with a material having a porous internal structure, is absorbed by it. This process causes the water to penetrate the capillary network, leading to the material becoming saturated and, consequently, impairing its properties. For example, cyclic changes in moisture content cause swelling or shrinkage of materials, which can ultimately reduce their durability [1,2,3]. Water also exerts a similar destructive effect on building materials when it contains dissolved salts [4], or when the water present in the capillaries freezes [3,4]. Furthermore, the mere filling of pores with water negatively affects materials, reducing their thermal insulation properties. In all these cases, the intensity of the processes depends on the transport of capillary water.

The capillary rise process is most often characterized by the absorption coefficient ($A_{w}$), which is defined as the ratio of the mass of water absorbed by the sample ($m_{w}$) to the surface area of the cross-section submerged in water (A), and the square root of time ($\sqrt{t}$). This type of characterization is correct when the relationship between the mass of water and time is proportional to the square root of time. However, in many cases, deviations from this relationship are observed, requiring more accurate measurements of the water mass to faithfully capture the true course of the phenomenon.

The cause of these deviations is often dynamic effects resulting from the changing contact angle at the water–air interface [5]. In the case of cementitious materials, it is believed that the source of these deviations is their hygroscopic nature [6,7,8]. In such situations, more complex models are required to describe the process accurately. However, the measurement principle remains unchanged. It consists of recording the mass increase in the sample, whose lower surface remains in contact with water, allowing capillary rise of the liquid against gravity.

The basic approach is the gravimetric method [9], in which the mass is measured by periodically removing the sample from the water at specified intervals and weighing it. Although the simplicity of this method is its advantage, a significant disadvantage is the measurement error caused by the necessity of removing the sample for weighing.

A modification of the standard method is the continuous gravimetric method [10,11]. In this approach, the sample is suspended on a scale while maintaining contact with water. Contact is achieved by immersing one surface of the sample to a depth of a few millimeters. The mass of the sample is then automatically recorded at high frequency. As shown in this article, the disadvantage of this approach is the inaccuracy of measurements in the initial phase. This results from the surface tension acting on the part of the sample submerged in water (about 2–3 mm), which changes the force acting on the sample depending on its moisture content. However, this process stabilizes after some time, and the changes in the scale readings reflect only the changes in the mass of water penetrating the sample.

There are also more advanced methods that can be used to determine the spatial distribution of moisture within a material. By integrating these distributions over the volume, mass changes over time can also be obtained. Such methods include isotopic techniques [12,13,14], nuclear magnetic resonance (NMR) methods [15,16,17], and X-ray based methods [18,19]. Although these techniques can provide detailed moisture distribution data, they have significant limitations. A major drawback is that they require complex instrumentation, and the samples used for analysis must typically be small in size.

In recent years, new methods for characterizing capillary rise in porous building materials have emerged, highlighting the importance and relevance of this issue. In [12], thermographic analysis was used to study capillary rise. This method involved thermographic measurements of the lateral surface of a sample immersed in water. From the apparent temperature, which depends on the level of moisture content, the mass of adsorbed water was determined, allowing the absorption coefficient to be calculated.

In the study presented in [20], a unilateral nuclear magnetic resonance (NMR) technique was used to determine the absorption coefficient. This method was tested on samples of brick, sandstone, mortar, and concrete, and the results were compared with those obtained using the traditional gravimetric method with continuous measurement.

An interesting technique for measuring the amount of water adsorbed by capillary rise was presented in [21]. The method used a vessel system in which the displacement of the plunger in one of the vessels was measured, allowing the relationship between the plunger movement and the moisture content of the sample to be monitored.

In turn, the authors of [22] investigated capillary rise in brick masonry using specially designed capacitive and resistive sensors. The results showed a strong correlation between the mass of water absorbed and changes in capacitance, as well as the ability to monitor the capillary rise process in real time.

The limited applicability of some of the methods presented, along with the development of new techniques, indicates that the phenomenon of capillary rise of water in building materials is still not fully understood, and the need for precise measurements remains relevant. Furthermore, the validity of addressing this issue is confirmed by the fact that certain anomalies in the capillary rise measurements have been reported in the literature, and these are still the subject of intensive research [8,9,10]. It appears necessary to develop alternative methods, particularly non-contact ones, that will support a better understanding of the nature of this process.

This study proposes an innovative and precise approach for measuring capillary rise. The method is based on tracking the displacement of a reference point on the sample surface during the capillary absorption process. Measurements are performed in a non-contact manner using Digital Image Correlation (DIC).

Unlike traditional gravimetric methods, which suffer from significant errors in the initial measurement phase due to surface tension forces, the proposed method eliminates this problem. As a result, it provides higher accuracy and improved temporal resolution. Incorporating the phenomenon of moisture-induced expansion of the material enabled a quantitative correlation between the recorded displacements and the mass of absorbed water. This represents a key innovation and the main contribution of this research to the development of moisture transport measurement methodologies in porous materials.

A literature review [23,24] shows that, while the effect of changes in liquid water on the deformation of porous building materials has been previously studied, the phenomenon of moisture expansion has been used for the first time as a tool to determine the amount of absorbed water. The proposed method opens new possibilities for the precise analysis of moisture-related processes. It also provides a solid foundation for further studies on the characterization and control of capillary rise in building materials. This may contribute to improving the durability and functionality of these materials.

## 2. Problem Statement

**Figure 1:**
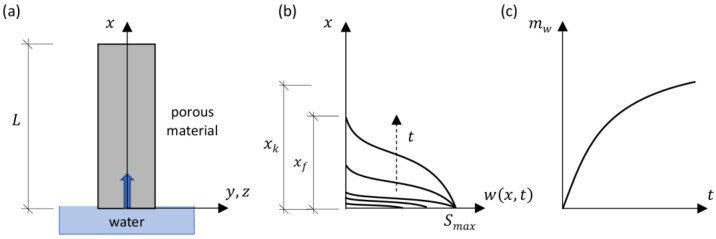
Capillary rise process: (**a**) source of moisture; (**b**) moisture content distribution along the specimen height; (**c**) evolution of adsorbed water mass over time.

As a result of capillary rise (Figure 1a), the saturation distribution [–] changes along the height of the sample (Figure 1b). At the contact surface between the material and water, this process causes a sharp increase in saturation to its maximum value, $S_{max}≅1$ [–]. Simultaneously, the moisture front, $x_{f}$, begins to move upward. By integrating the saturation distribution (Figure 1b), multiplied by the maximum mass moisture content, $w_{max}$ [kg/m^3^], over the volume, the mass of water adsorbed by the sample, $m_{w}$ [kg], can be determined (Figure 1c).

**Figure 2:**
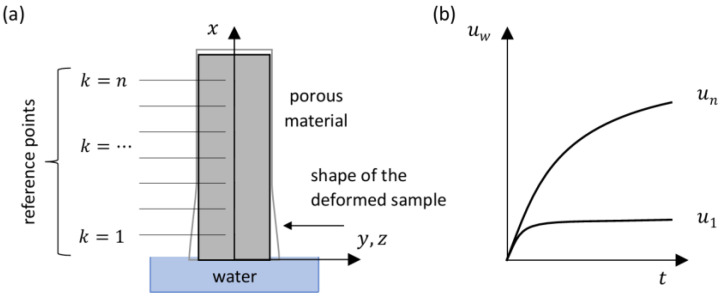
Moisture-induced expansion process: (**a**) schematic representation; (**b**) displacement of selected reference points on the specimen surface.

At the same time, the swelling process causes the deformation of the sample. Figure 2 shows a schematic representation of the process along with the displacements of the reference points, $u_{k}$, on the surface.

The time-varying domain of the described process progressively encompasses successive reference points. Once the front of adsorbed water passes through the analyzed points, their displacements stabilize, making them unsuitable for further analysis. Therefore, the reference point used in the analysis should be located as high as possible in order to extend the duration of the observation. In addition, a considerable distance between the moving front and the measurement point reduces the effect of transverse deformations at the bottom of the sample on its vertical displacements. This is of crucial importance when a beam model is applied in mechanical analysis.

## 3. Mathematical Model for Determining the Mass of Adsorbed Water from Displacement Measurements

To relate the capillary rise process to the deformation of the sample, a beam model was used. We analyze the displacements of a beam with length L [m] and a symmetrical cross section with area A [m^2^], subjected only to a water saturation field, $S\left(x,y,z,t\right)$, where the saturation, S, is defined as the ratio of the current gravimetric moisture content, $w\left(x,y,z,t\right),$ to the maximum (saturated) gravimetric moisture content, $w_{max}$, i.e., $S=w/w_{max}$. This ratio varies in space and time. Using the linear elasticity theory for beam systems, the following equations are available:

− Equilibrium equations:
  (1) $$\int_{A}\sigma dA=N=0,$$
  (2) $$\int_{A}\sigma \cdot zdA=M_{y}=0,$$
  (3) $$\int_{A}\sigma \cdot ydA=M_{z}=0,$$

− Physical equations:
  (4) $$\sigma =E\cdot \left(\varepsilon -\varepsilon_{w}\right),$$
  (5) $$\varepsilon_{w}=\alpha_{w}\cdot ∆S=\alpha_{w}\cdot \left(S-S_{0}\right)=\frac{\alpha_{w}}{w_{max}}\cdot \left(w-w_{0}\right),$$

− Geometric equations (Bernoulli’s hypothesis of plane cross sections):
  (6) $$\varepsilon (x,y,z)=c_{1}(x)+c_{2}(x)\cdot y+c_{3}(x)\cdot z,$$
  where σ—the stress [Pa]; ε—the deformation [−]; $\varepsilon_{w}$—the moisture deformation [−]; $\alpha_{w}$—the coefficient of moisture expansion [−]; S and $S_{0}$—the current and initial water saturation, respectively [−]; w and $w_{0}$—the current and initial gravimetric moisture content, respectively [kg/m^3^]; $w_{max}$—the maximum (saturated) gravimetric moisture content [kg/m^3^].

By solving the system of Equations (1)–(6) under the assumption of an axially symmetric moisture field, we obtain the following result:

(7) $$c_{2}\left(x,t\right)=c_{3}\left(x,t\right)=0,\;\varepsilon \left(x,t\right)=c_{1}\left(x,t\right)=\frac{\int_{A}\alpha_{w}\cdot ∆S\left(x,y,z,t\right)dA}{A}.$$

The moisture expansion coefficient is, in principle, a function of the material’s moisture content. However, in the case of capillary rise, the pores become fully saturated with water, resulting in a constant moisture state. Under such conditions, it is justified to assume a constant value of the moisture expansion coefficient, corresponding to full saturation of the material.

Naturally, local heterogeneities in moisture distribution and the presence of partially saturated zones may occur. Nevertheless, these factors are not expected to significantly affect the variability of the moisture expansion coefficient.

The displacement at the analyzed point, x, can be obtained by integrating the strain equation along the x-direction within the limits $\langle 0,x\rangle$. Taking into account the boundary condition,

(8) $$u\left(x=0,t\right)=0$$

we obtain the following equation:

(9) $$u\left(x,t\right)=\int_{0}^{x}\varepsilon \left(\xi ,t\right)d\xi .$$

In the event that $x=x_{k}>x_{f}$ (ref. to Figure 1 and Figure 2), and assuming that the moisture expansion coefficient remains constant, the displacement of point k can be expressed as follows:

(10) $$u_{k}\left(t\right)=\alpha_{w}\cdot \int_{0}^{x_{f}}∆S_{mean}\left(\xi ,t\right)d\xi ,$$

where

(11) $$∆S_{mean}\left(x,t\right)=\frac{\int_{A}∆S\left(x,y,z,t\right)dA}{A}.$$

Let us proceed to an analysis of the relationship between the change in the sample’s mass and the change in the distribution of moisture within the sample. The mass increase can be calculated by integrating the moisture increase over the volume of the entire beam:

(12) $$∆m_{w}\left(t\right)=w_{max}\cdot \int_{V}∆S\left(x,y,z,t\right)dV=\;w_{max}\int_{0}^{L}\int_{A}∆S\left(x,y,z,t\right)dAdx=A\cdot w_{max}\cdot \int_{0}^{L}∆S_{mean}\left(x,t\right)dx.$$

In the event that $x_{f}<L$, the weight gain is

(13) $$∆m_{w}\left(t\right)=A\cdot w_{max}\cdot \int_{0}^{x_{f}}∆S_{mean}\left(\xi ,t\right)d\xi .$$

Finally, inserting Equation (13) into (10), we obtain a relation that relates the increase in the mass of the sample to the displacement of the points x in the form of:

(14) $$u\left(x_{k},t\right)=\frac{\alpha_{w}}{A\cdot w_{max}}\cdot ∆m_{w}\left(t\right)\;∆m_{w}\left(t\right)=\frac{A\cdot w_{max}}{\alpha_{w}}\cdot u\left(x_{k},t\right).$$

From the above equation, it follows that, in order to correlate the displacement of the analyzed point with the increase in mass, it is necessary to know the moisture expansion coefficient of the tested material. This coefficient can be determined by measuring the displacement and the mass of the sample at the same time. During the test, this can be accomplished by measuring the mass of the sample immediately after the test is completed. The coefficient of expansion can then be calculated from the equation:

(15) $$\alpha_{w}=A\cdot w_{max}\cdot \frac{u\left(x_{k},t_{end}\right)}{∆m\left(t_{end}\right)}.$$

The relationship between the change in mass and the displacement of point k is given by the following equation:

(16) $$∆m_{w}\left(t\right)=u_{k}\left(t\right)\cdot \frac{∆m\left(t_{end}\right)}{u_{k}\left(t_{end}\right)}.$$

In the proposed method, this relationship serves as the basis for the determination of the mass of adsorbed water on the basis of displacement measurements.

## 4. The Experiment

Sample Preparation

**Figure 3:**
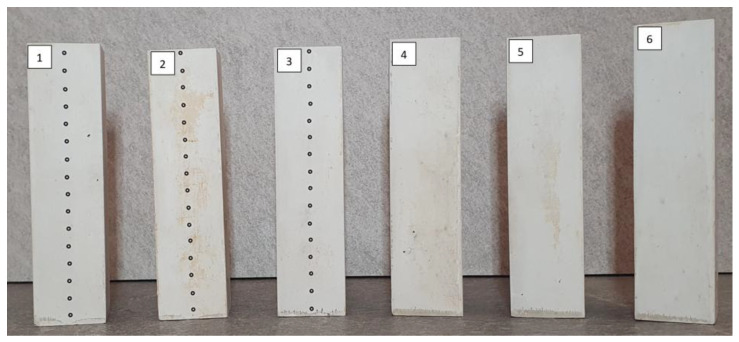
Prepared gypsum specimens: samples 1–3 used for DIC measurements; samples 4–6 used for gravimetric measurements.

The samples tested were prismatic in shape, with a square base of 40 mm per side and a height of approximately 160 mm. A total of six samples were prepared (Figure 3), with three for each measurement method. All samples were made from a single batch.

The specimens were conditioned in a climate chamber at 50% relative humidity (RH) for two weeks after the gypsum set and the specimens were demoulded. They were then dried at a temperature of 50 °C. Special measuring markers (designed for the measuring system used) were placed vertically along the center of one of the side surfaces of the samples designated for optical testing. The water and the samples used in the experiment were conditioned in the test room for two days prior to the experiment. This ensured thermodynamic equilibrium between the environment, the samples and the water used during the test.

### 4.1. Main Research

Measurement Bench

**Figure 4:**
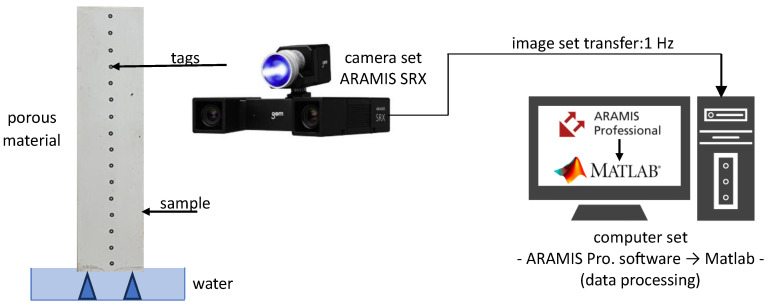
Schematic diagram of the DIC experimental setup.

The measurement setup for the method proposed in this study consisted of components of the Digital Image Correlation (DIC) system, which allows displacement measurements, and a water container equipped with three point supports for the sample, which allows water absorption through the bottom surface of the sample. A schematic of the measurement setup is shown in Figure 4.

The displacement measurements were performed using the GOM Aramis SRX Digital Image Correlation (DIC) system. This system employs a stereo-camera configuration that enables full-field, non-contact measurement of displacements and deformations with high spatial resolution. The system was equipped with two high-resolution cameras, each providing a maximum resolution of 4096 × 3068 pixels. For the purpose of the present study, the height of the image frame was limited to one-sixth of the full sensor area, and the cameras were rotated by 90 degrees. As a result, an effective resolution of 480 × 4096 pixels was achieved in the measurement area, which allowed precise tracking of displacements along the height of the tested samples.

The cameras were mounted on a GOM 180/12M measuring bar(GOM GmbH, Braunschweig, Germany)with a working space of 260 mm × 200 mm. The mounting system ensured stable positioning of the cameras and proper alignment during the experiment. The system calibration was carried out using a certified calibration panel provided by the manufacturer, following the recommended procedure to ensure measurement accuracy.

To track the displacements, original circular, high-adhesion, thermally stable markers with a diameter of 0.8 mm were applied vertically along the central axis of one of the side surfaces of each sample. These markers, specifically designed for use with the GOM system, provided excellent contrast and ensured reliable tracking throughout the experiment.

The global coordinates of the reference points were determined using GOM Correlate Professional 2021 software, which offers advanced tools for image correlation, calibration, and displacement measurement. For further data processing, including the conversion of displacements into the mass of absorbed water and detailed analysis of the results, proprietary software developed in the MATLAB 2024 (MATLAB 2024, MathWorks Inc., Natick, MA, USA)environment was utilized. This approach allowed for full control of the data processing workflow and ensured compatibility with the mathematical model proposed in this study.

The displacement data were recorded continuously with a frequency of 1 Hz for a total of 60 min per sample.

Course of the Study

The tested sample was placed on the point supports and the container was filled with water so that the sample was immersed to a height of approximately 2 mm. During the initiated capillary rise process, the Aramis system continuously recorded (at a frequency of 1 Hz) the displacements of the markers on the lateral surface. The measurement for each sample took 60 min. This produced displacement curves for each point, allowing analysis of the capillary rise process.

Each prismatic sample was weighed immediately before the start of the test and immediately after the end of the test. This allowed calculation of the total amount of water absorbed during the test. This information was essential in applying Equation (15) to relate the recorded displacements to the amount of water adsorbed at a given time.

### 4.2. Gravimetric Measurements

Measurement Bench

**Figure 5:**
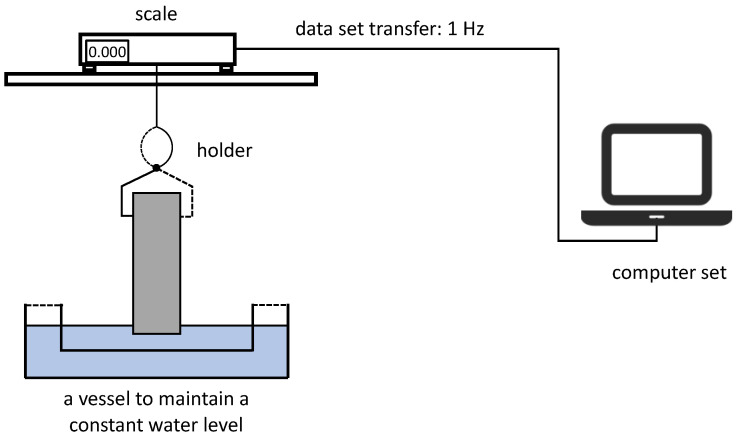
Schematic diagram of the gravimetric measurement setup.

The measurement setup for the gravimetric method consisted of a scale mounted on a special stand and a container that maintained a constant water level during the test. The scale, which was connected to a computer, enabled the continuous recording of the current measured mass. It also included a suitable holder for suspending the sample to be tested. A schematic of the measurement setup is shown in Figure 5.

Course of the Study

The sample to be tested was suspended in the holder below the scale. Using the adjustable stand, the scale was lowered until the lower surface of the beam touched the surface of the water in the container below. The capillary rise process caused a change in the mass of the sample, which was continuously recorded (at a frequency of 1 Hz) in the computer memory. This process produced a graph of the increase in mass during the test.

As with the previous method, each sample was weighed immediately before and after the test. This allowed validation of the results obtained with this method.

## 5. Uncertainty Analysis

### 5.1. Measurement Uncertainty

As follows from Equation (14), the mass of absorbed water is calculated based on the displacements of reference points, the cross-sectional area, the moisture content at full saturation, and the moisture expansion coefficient. The measurement uncertainties of these quantities contribute to the overall uncertainty of the determined mass. However, when Equation (15) is taken into account, the water mass calculated using Equation (16) no longer depends on the aforementioned values, but solely on the displacements of the reference points and the ratio between displacement and mass at the end of the experiment. Therefore, the uncertainty of the computed mass depends only on the uncertainty of the displacement measurement and the final mass measurement. According to the law of propagation of uncertainty, it is given by:

(17) $$un\left(∆m\right)=\sqrt{{\left(\frac{\partial ∆m}{\partial {∆m}_{end}}un(m)\right)}^{2}+{\left(\frac{\partial ∆m}{\partial u_{end}}un(u)\right)}^{2}+{\left(\frac{\partial ∆m}{\partial u}un(u)\right)}^{2}}.$$

That is:

(18) $$un\left(∆m\right)=\frac{{∆m}_{end}}{u_{end}}\sqrt{{un(u)}^{2}+\left(\frac{{un(u)}^{2}}{{u_{end}}^{2}}+\frac{{un(m)}^{2}}{{{∆m}_{end}}^{2}}\right)\cdot {u_{k}}^{2}}.$$

where $un\left(∆m\right)$, $m_{end}$, un(m), $u_{end}$, and un(u) denote, respectively: the uncertainty of the determined mass, the final absorbed water mass, the uncertainty of the measured final mass, the final displacement, and the uncertainty of the measured displacement.

Assuming that the accuracy of the measuring instruments δx is treated as their limit uncertainty, the standard uncertainty can be obtained from the relation:

(19) $$un\left(x\right)=\frac{\delta x}{\sqrt{3}},$$

where $un\left(x\right)$ is the standard measurement uncertainty corresponding to the standard deviation for a uniform distribution of variable x. Applying this relationship to the displacement and final mass measurements, we obtain:

(20) $$un\left(u\right)=\frac{e_{u}\cdot u_{k}}{\sqrt{3}},\;un\left(m\right)=\frac{e_{m}\cdot {∆m}_{end}}{\sqrt{3}},$$

where $e_{u}$ and $e_{m}$ are the ratios of the instrument accuracy to the measured displacement and final mass values, respectively.

Considering Equation (20), the relative measurement uncertainty of the determined mass can be calculated as:

(21) $${un}_{r}\left(∆m\right)=\frac{un\left(∆m\right)}{∆m}=\frac{1}{\sqrt{3}}\sqrt{{e_{u}}^{2}\cdot \left(1+{\left(\frac{u_{k}}{u_{end}}\right)}^{2}\right)+{e_{m}}^{2}}.$$

### 5.2. Systematic Error

The application of the bar model introduces a systematic error in determining the elongations caused by capillary water uptake. Displacements calculated using the one-dimensional (1D) bar model differ from those obtained with a three-dimensional (3D) mechanical model. This discrepancy also affects the uncertainty of the determined mass. To estimate the relative error between both models as a function of the position of the advancing moisture front, the following formula was used:

(22) $${∆}_{mod}=\left|\frac{w_{3D}-w_{1D}}{w_{1D}}\right|\cdot 100\%,$$

where $w_{1D}$ and $w_{3D}$ represent the elongations in millimeters for the bar model and the three-dimensional model, respectively.

The analysis of the obtained differences was conducted for various heights of the moisture front, assuming that the transition zone between the wet and dry parts of the material is very narrow (a sharp front), i.e., the moisture content changes abruptly. This assumption represents the most unfavorable case in terms of differences between the models. The analysis was carried out for a cylindrical bar with a diameter of 4 cm and a height of 16 cm. Cases were considered in which the sharp moisture front was located at nine different heights—ranging from 0 cm to 8 cm in 1 cm increments.

**Figure 6:**
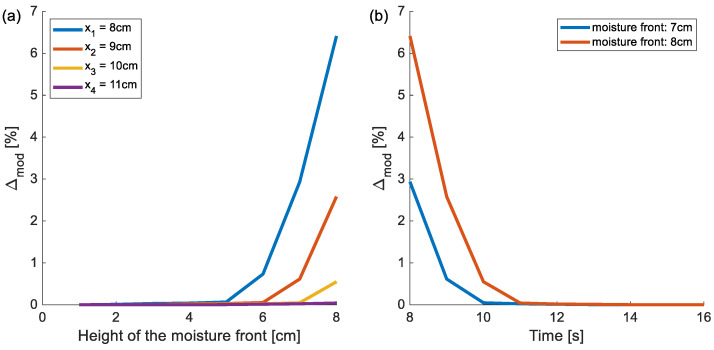
Relative error between the 1D bar model and the 3D mechanical model as a function of (**a**) moisture front height and (**b**) reference point position along the specimen.

The plot in Figure 6a shows that the relative difference decreases with increasing position of the reference point, and for points located above 11 cm, it becomes very small. In the analyzed case, when the moisture front reaches half the height of the specimen, the bar model provides sufficient accuracy for reference points located above 11 cm (Figure 6b).

### 5.3. Total Uncertainty

The previous sections show that the uncertainty in the measured displacements results from two main sources: the inaccuracy of the measuring equipment and the systematic error of the adopted mechanical model. In this case, the total displacement uncertainty can be expressed as follows:

(23) $$un\left(u\right)=\sqrt{{\left({un}_{a}\left(u\right)\right)}^{2}+{\left({un}_{s}\left(u\right)\right)}^{2}}$$

where ${un}_{a}\left(u\right)$ and ${un}_{s}\left(u\right)$ are the uncertainties arising from instrument inaccuracy and the systematic modeling error, respectively. Assuming

(24) $${un}_{a}\left(u\right)=\frac{e_{a}\cdot u_{k}}{\sqrt{3}},\;{un}_{s}\left(u\right)=\frac{e_{s}\cdot u_{k}}{\sqrt{3}},$$

where $e_{a}$ and $e_{s}$ denote the relative instrument inaccuracy and the relative systematic error, respectively, the relative uncertainty of the determined water mass (as given by Equation (21)) ultimately takes the following form:

(25) $${un}_{r}\left(∆m_{w}\right)=\frac{un\left(∆m_{w}\right)}{∆m_{w}}=\frac{1}{\sqrt{3}}\sqrt{\left({e_{a}}^{2}+{e_{s}}^{2}\right)\cdot \left(1+{\left(\frac{u_{k}}{u_{end}}\right)}^{2}\right)+{e_{m}}^{2}}.$$

The accuracies of the displacement and mass measurement instruments used in the experiments were $\delta_{a}=0.0013\;mm$ and $\delta_{m}=0.02\;g$. If the final displacement and absorbed water mass were $u_{end}=0.056\;mm$ and ${∆m}_{end}=28.99\;g$, and, for example, the intermediate displacement was $u_{k}=0.03\;mm$, then the relative measurement errors are $e_{a}=4.3\%$ and $e_{m}=0.1\%$. If the systematic error is assumed to be $e_{a}=0.1\%$, then the relative uncertainty of the absorbed water mass at that moment would be ${un}_{r}\left(∆m\right)=2.8\%$. At the end of the experiment, the relative uncertainty decreases to ${un}_{r}\left(∆m\right)=1.9\%$. This level of uncertainty is considered satisfactory.

## 6. Applicability Range of the Model

Given that the proposed method is novel, it is necessary to define the class of materials and their properties that allow its application.

The uncertainty analysis shows that the uncertainty in the determined mass of absorbed water depends not only on the accuracy of the measuring instruments but also on the magnitude of the measured displacements. These displacements, in turn, depend on the moisture expansion coefficient and the amount of water absorbed by the tested specimen—which corresponds to the height to which the material can draw water. The decisive factor is the product of these two quantities, as shown by Equation (10), assuming that $∆S_{mean}=1$:

(26) $$u_{k}\left(t\right)=\alpha_{w}\cdot x_{f}\left(t\right)\cdot 1.$$

To ensure that the uncertainty of the obtained results remains acceptable, for instruments with a measurement accuracy comparable to those used in this study (δu=0.0013 mm, δm=0.002 g), the product $\alpha_{w}\cdot x_{f}$ should not be less than 0.05 mm. This condition can be met by most construction materials. It should be noted, however, that the height of the water absorption front is a time-dependent function, i.e., $x_{f}=x_{f}\left(t\right)$. Therefore, meeting this requirement for different materials may require either longer or shorter durations for the test, depending on the specific absorption characteristics of the material.

## 7. Results

### 7.1. Displacement Method

**Figure 7:**
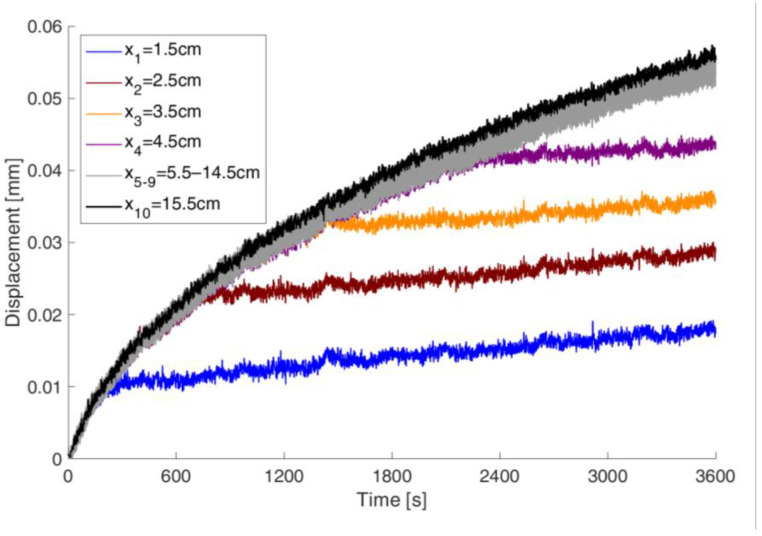
Example of recorded displacement curves for 10 reference points along the specimen height during the capillary rise process.

Three samples (#1–3) were tested using the proposed method. Each test took 60 min. The measurements provided displacement data for the reference points during the capillary rise process. A sample graph of the recorded displacements for 6 reference points is shown in Figure 7. The first reference point was located 1.5 cm above the water surface, and the subsequent points were placed every 1 cm up the sample. The last point was positioned 15.5 cm above the water surface. The notation $x_{k}=5.5-14.5$ cm refers to a series of 10 curves that show only slight differences within this range.

It can be seen that the front of the absorbed moisture passed through the lower points during the 60 min period. To apply the model presented earlier, such a point should remain above the moisture front throughout the test. Therefore, the highest points on the samples were selected for further analysis. These data were also smoothed using a spline function.

**Figure 8:**
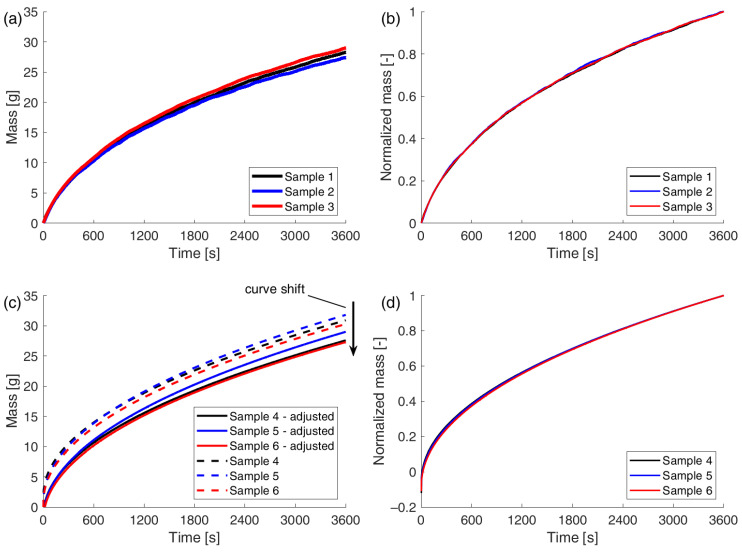
Evolution of absorbed water mass: (**a**) absolute mass obtained from displacement method; (**b**) normalized mass obtained from displacement method; (**c**) absolute mass from gravimetric method after correcting for additional forces: (1) surface tension force; (2) buoyant force; (3) adhesive liquid layer weight; (**d**) normalized mass obtained from gravimetric method.

The masses of water absorbed during the entire measurement period were as follows: $m_{1}=28.259\;g$, $m_{2}=27.369\;g,\;$ and $m_{3}=28.979\;g$ for samples 1, 2, and 3, respectively. Equation (15) was used to convert the displacements of the highest points on the samples into the mass of water absorbed. The data obtained in this way are presented in the form of graphs in Figure 8a.

Although all the tested samples came from the same batch, their cross-sectional dimensions differed slightly. As a result, even when water was drawn to the same height, the mass of absorbed water varied. To allow for an objective comparison of results across different samples, the masses were normalized using the following formula:

(27) $$\tilde{m}\left(t\right)=\frac{m_{w}(t)}{m_{w}(t=3600\;s)}.$$

The result of this transformation is shown in Figure 8b.

An analysis of Figure 8b shows that there is almost no overlap between the results for all samples.

### 7.2. Gravimetric Method

Three samples (#4–6) were tested using the gravimetric method. Each test took 60 min. The measurements provided records of the masses of absorbed water registered by the scale. As mentioned in the description of the experiment, weighing the samples before and after the test enabled the validation of the results. Significant differences were observed between the final masses of water recorded during the test and the masses obtained from the control measurements. The results of this comparison are presented in Table 1.

These discrepancies arise from the fact that the scale does not indicate the true mass of absorbed water. The measurement is affected by errors resulting from the action of the following:

− The surface tension force (acting downward);
− The buoyancy force (acting upward) in the early stage, when the bottom of the sample is slightly below the water surface;
− The force due to the weight of a thin layer of liquid adhering to the bottom surface of the sample as a result of adhesion (acting downward) in the later stage, when the bottom of the sample is slightly above the water surface.

**Figure 9:**
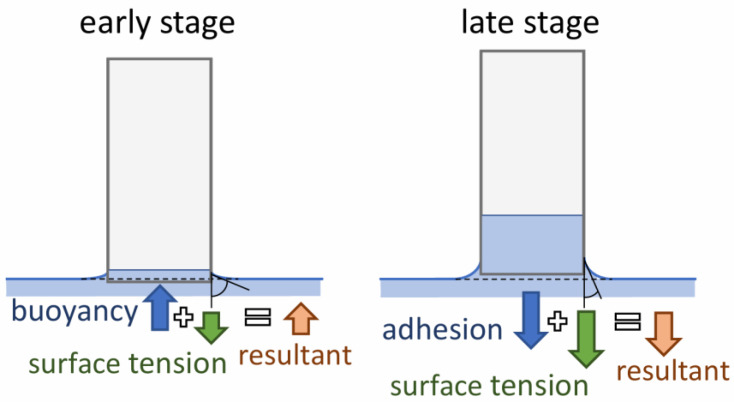
Additional forces acting on the specimen during the gravimetric test: surface tension force; buoyant force; adhesive liquid layer weight.

Forces acting upward are recorded by the scale as a reduction in mass, while downward-acting forces cause the measured mass to be higher than the actual value. At the beginning of the test, the bottom of the sample is positioned just below the water surface. However, as the test progresses and water is absorbed by the sample, the water level in the container gradually decreases. In the final phase, the water level may even fall below the bottom of the sample (see Figure 9). The additional mass of water that remains “suspended” from the sample, combined with the surface tension forces, results in the measured mass at the end of the test being greater than the true value.

To correct the results obtained, the mass differences shown in Table 1 should be subtracted from the recorded data. On the graph shown in Figure 8c, this will be visible as a downward shift of the curves.

This figure also shows that the corrected mass values become positive only after several seconds, indicating that the force acting on the sample due to surface tension changes with time. The value of this force will not stabilize until the lower layers of the sample are fully saturated and thermodynamic equilibrium is reached at the water–sample interface.

As mentioned earlier, samples may differ slightly from each other. In order to compare the results obtained for them, the masses were normalized according to Equation (16) and the plots are shown in Figure 8d.

The graphs show that the process of capillary rise is almost identical in the three samples analyzed, with the exception of the initial phase.

### 7.3. Analyzed Methods Comparison

All the tested samples are slightly different from each other. This results in each sample absorbing a slightly different mass of water at any given time. However, the differences are minimal, as shown in Table 2.

**Figure 10:**
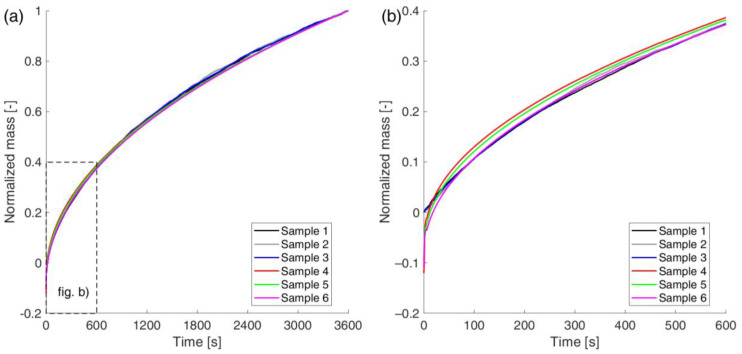
Normalized absorbed water mass for all specimens: (**a**) full time range; (**b**) first 10 min of the experiment.

In order to compare the results obtained with the two methods for samples that are not perfectly identical, it was decided to compare the normalized masses, expressed as a ratio from 0 to 1. Their evolution over time is shown in the form of graphs in Figure 10. Analyzing this graph, it can be seen that both methods converge well, but only after about 10 min.

**Figure 11:**
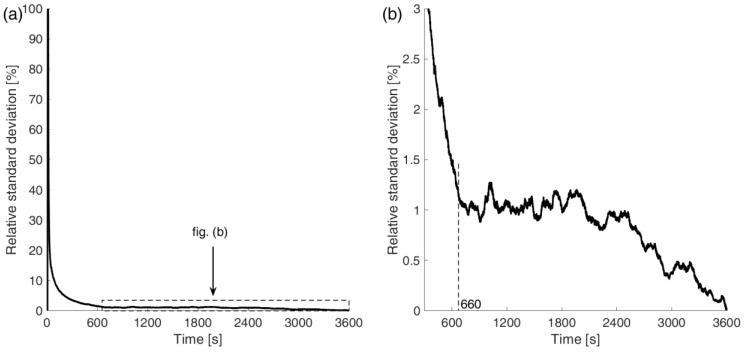
Relative standard deviation of normalized absorbed water mass for all specimens as a function of time.

To objectively determine the time after which the two methods converge, the relative standard deviation of the absorbed water mass at a given time for all six samples was plotted in Figure 11. It was calculated according to the following formula:

(28) $$\sigma_{rel}\left(t\right)=\frac{\sqrt{\frac{1}{6}\sum_{i=1}^{6}{\left[{\tilde{m}}_{i}\left(t\right)-\langle \tilde{m}\rangle (t)\right]}^{2}}}{\langle \tilde{m}\rangle (t)}.$$

where ${\tilde{m}}_{i}\left(t\right)$—normalized mass of the i-th sample at time *t*; $\langle \tilde{m}\rangle (t)$—arithmetic mean according to the following formula:

(29) $$\langle \tilde{m}\rangle (t)=\frac{1}{6}\sum_{i=1}^{6}{\tilde{m}}_{i}\left(t\right).$$

Analyzing the graphs in Figure 11, it can be seen that the relative standard deviation takes very high values at the beginning of the process. Only after 11 min does it stabilize at a low level, below 1.5%. It can be concluded that both methods initially show significantly different values for the absorbed water. This is most likely due to the previously described phenomenon of the change in surface tension at the sample–water interface, which causes the gravimetric method to give incorrect readings at the beginning. However, as the process progresses, both methods show very high convergence and give very similar values.

### 7.4. Evaluation of the Suitability of the Proposed Method

**Figure 12:**
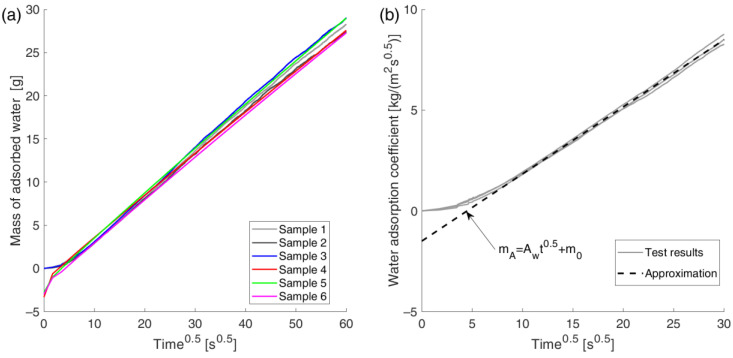
Absorbed water mass as a function of the square root of time: (**a**) mass evolution; (**b**) water absorption coefficient determined using the displacement method.

Analysis of the graph of the mass of water absorbed as a function of the square root of time (see Figure 12) shows that, except for the initial phase of the test, the relationship is linear for the material tested. Therefore, this process can be well described by a diffusion model of capillary flow with dynamic effects during the initial phase (see Figure 12b).

To evaluate the applicability of the proposed method for studying the capillary rise process, it was decided to use it to determine the water adsorption coefficient ${(A}_{w})\;$ in the mentioned model.

As shown in Section 5.2, the initial part of the gravimetric data is affected by measurement errors. Therefore, it was decided not to include these data in the calculation of the water adsorption coefficient. For both methods, only data for times t ≥ 660 s were considered.

The determined coefficient is directly the slope of the straight line of the function (see Figure 12b):

(30) $$m_{A}=\frac{m_{w}(t)}{A}=A_{w}\cdot \sqrt{t}+m_{0}$$

where $m_{w}$—water mass [kg]; $A_{w}$—adsorption coefficient [kg/(m^2^s^0.5^)]; t—time [s]; $m_{0}-m_{A}$ value for t=0; A—area of contact between sample and water [m^2^].

**Figure 13:**
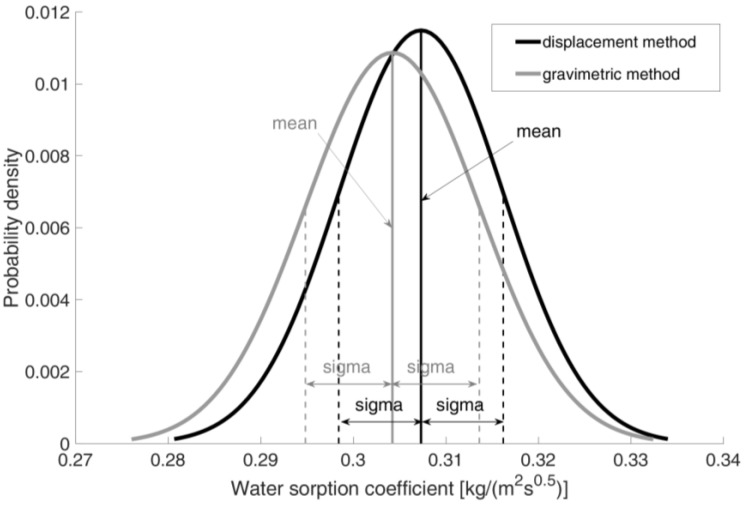
Probability density distribution of water absorption coefficient results obtained with the displacement method and the gravimetric method.

The coefficient $A_{w}$ was determined using the least squares method, where the Levenberg–Marquardt algorithm was used to minimize the objective function. The results obtained for each dataset are presented in Table 3 and Figure 13.

A review of Table 3 and the graph in Figure 13 reveals that the obtained results exhibit minimal variation. The relative standard deviations are approximately 3%. Furthermore, the results are consistent with data from the literature [11,15], which range from 0.290 to 0.306 [kg/(m^2^ s^0.5^)].

It is noteworthy that the discrepancies in the mean values yielded by the two methods are less than one standard deviation. It can thus be concluded that both methods yield results that are essentially identical. This lends further support to the applicability of the proposed method for such studies, at least to the same extent as the gravimetric method with continuous measurement.

## 8. Conclusions

The authors propose and demonstrate a method that allows the capillary rise process to be studied using optical measurements. Furthermore, a comparison was conducted with the most commonly utilized gravimetric method.

In conclusion, the results of the conducted research allow us to draw several key conclusions:

- The proposed method, based on tracking the displacements of points on the surface of the sample, enables the analysis of the capillary rise process in building materials.
- The efficacy of the proposed methodology was validated through a comparative analysis with the most prevalent gravimetric approach. The results of the statistical analysis demonstrated that both methods yielded nearly identical outcomes, with a difference of approximately 1% between the average water adsorption coefficients determined by both methods.
- As has been shown, the gravimetric method is not an appropriate means of analyzing the initial stages of the capillary rise process. In contrast, the proposed method permits such an analysis.
- The presented measurement method allows for the tracking of displacements across the entire height of the sample. Once the mathematical model has been further developed, it will be possible to measure not only the total absorbed water mass but also the moisture distribution within the sample during capillary rise. This will enable the verification of capillary rise models proposed in the literature.
- One advantage of the proposed method is that it is non-contact, which eliminates any influence on the course of the studied process. Furthermore, it is possible to study even large samples, which is challenging for many other methods.
- To the authors’ knowledge, this is the first method based on moisture-induced expansion that enables quantifying the amount of water absorbed during capillary rise in porous materials.

## Figures and Tables

**Table 1:** Final masses of absorbed water determined by gravimetric measurements: comparison between continuously recorded mass and post-test control weighing.

Sample No.	Final Mass of Water Recorded During the Test [g]	Mass of Water from Control Measurements [g]	Mass Difference [g]
4	30.89	27.57	3.32
5	31.79	28.99	2.80
6	30.27	27.28	2.99

**Table 2:** Masses of absorbed water after 60 min of testing for all specimens.

Sample No.		Mass of Absorbed Water [g]	Mean Value [g]	Relative Standard Deviation [%]
Displacement method	1	28.26	28.07	2.8
	2	27.37		
	3	28.98		
Gravimetrical method	4	27.57		
	5	28.99		
	6	27.28		

**Table 3:** Water absorption coefficient values determined using the displacement method and gravimetric method for all specimens.

Sample No.	Calculated $A_{w}$ Coefficient [kg/(m^2^ s^0.5^)]	Mean Value [kg/(m^2^ s^0.5^)]	Relative Standard Deviation [%]	Mean Value [kg/(m^2^ s^0.5^)]	Relative Standard Deviation [%]
1	0.308	0.307	2.9	0.306	2.7
2	0.298				
3	0.316				
4	0.297	0.304	3.1		
5	0.315				
6	0.301				

## Data Availability

The original contributions presented in this study are included in the article. Further inquiries can be directed to the corresponding author.
